# Non-Linear Device Head Coupling and Temporal Delays in Large Animal Acceleration Models of Traumatic Brain Injury

**DOI:** 10.1007/s10439-022-02953-w

**Published:** 2022-04-02

**Authors:** Andrew R. Mayer, Josef M. Ling, Declan A. Patton, David D. Stephenson, Andrew B. Dodd, Rebecca J. Dodd, Julie G. Rannou-Latella, Douglas H. Smith, Victoria E. Johnson, D. Kacy Cullen, Timothy B. Meier, Rachel E. Kinsler

**Affiliations:** 1 The Mind Research Network/Lovelace Biomedical Research Institute, Pete & Nancy Domenici Hall, 1101 Yale Blvd. NE, Albuquerque, NM 87106 USA; 2 Neurology Department, University of New Mexico School of Medicine, Albuquerque, NM 87131 USA; 3 Psychiatry Department, University of New Mexico School of Medicine, Albuquerque, NM 87131 USA; 4 Psychology Department, University of New Mexico School of Medicine, Albuquerque, NM 87131 USA; 5 Center for Injury Research and Prevention, Children’s Hospital of Philadelphia, Philadelphia, PA 19146 USA; 6 Department of Neurosurgery and Penn Center for Brain Injury and Repair, Perelman School of Medicine, University of Pennsylvania, Philadelphia, PA 19104 USA; 7 Department of Neurosurgery, Medical College of Wisconsin, Milwaukee, WI 53226 USA; 8 Department of Cell Biology, Neurobiology and Anatomy, Medical College of Wisconsin, Milwaukee, WI 53226 USA; 9 Department of Biomedical Engineering, Medical College of Wisconsin, Milwaukee, WI 53226 USA; 10 Enroute Care Group, U.S. Army Aeromedical Research Laboratory, Fort Rucker, AL 36362-0577 USA

**Keywords:** Dynamic, Head kinematics, Sensors, Angular velocity, Linear acceleration

## Abstract

Accurate characterization of head kinematics following an external blow represents a fundamental aspect of traumatic brain injury (TBI) research. The majority of previous large animal studies have assumed an equivalent relationship between the device delivering the impulsive load and subsequent head kinematics rather than performing direct measurement (sensors or videography). The current study therefore examined factors affecting device/head coupling kinematics in an acceleration TBI model. Experiment 1 indicated ~ 50% reduction in peak angular velocity for swine head relative to the device, with an approximate doubling in temporal duration. The peak angular velocity for the head was not significantly altered by variations in restraint device (straps vs. cables), animal positioning or body mass. In Experiment 2, reducing the impulsive load by 32% resulted in only a 14% reduction in angular velocity of the head (approximately 69% head/device coupling ratio), with more pronounced differences qualitatively observed for angular momentum. A temporal delay was identified in initial device/head coupling, potentially a result of soft tissue deformation. Finally, similar head kinematics were obtained regardless of mounting the sensor directly to the skull or through the scalp (Experiment 3). Current findings highlight the importance of direct measurement of head kinematics for future studies.

**Supplementary Information:**

The online version contains supplementary material available at 10.1007/s10439-022-02953-w.

## Introduction

Dynamic rotational acceleration and deceleration of the head has been recognized as a fundamental aspect of traumatic brain injury (TBI) throughout six decades of experimental work,^18^ and represents a common factor across most human injury scenarios (motor vehicle crashes, blast injury, fall, assault, sporting collision, *etc*.). Head kinematics (i.e., linear/angular velocity and acceleration) have been associated with both diffuse axonal injury and blood brain barrier dysfunction, and are frequently used to design critical injury thresholds in finite element models that span multiple species.^2,9,19^ However, the majority of previous large animal acceleration studies and computational models have typically measured the initial impulsive load from the device and assumed an equivalent transfer of energy to the head, which may result in scaling inaccuracies across species if the transfer of energy is not truly equivalent.^2,9,34,35^

Specifically, only a handful of large animal studies have utilized head-mounted sensors^1,11,16,18,25,26^ or high-speed video capture^16,23,24^ to quantify head kinematics during acceleration injury (reviewed in Ref. 18). Previous cadaver studies have used various methods for mounting sensors to the skull (see Supplemental Table 1), ranging from directly affixing a sensor to the skull^36^ to mounting the sensor to the skull through the scalp^37^ among other techniques. The impact of these variations in sensor mounting techniques on measured head kinematics has not been established. Moreover, previous sensor studies also intentionally varied the impulsive load across different head impact models: drop (helmeted and unhelmeted),^26^ sled,^16^ linear impactors^11,25^ and captive bolt guns.^1^ In contrast, a recent study by our group^18^ using the HYGE device established coefficients of variation (COV) that ranged between 1 and 2% for the device and between 8 and 12% for head kinematic parameters, both of which are generally considered to be within the acceptable ranges in terms of reproducibility.^7,15^ However, results also indicated unexpected large differences in angular velocity between the initial impulsive load as measured at the device (mean peak angular velocity of 250.51 rad/s) and a triaxial sensor mounted to the head (mean peak angular velocity of 130.22 rad/s), with an approximate doubling in temporal duration for head kinematics.

There are multiple experimental factors that influence the transfer of energy between the impulsive load and the head (i.e., device to head coupling), as well as potentially confounding the measurement of true head kinematics. For example, individual differences in animal morphology (snout length/width, presence of prominent dental structures such as mature canine teeth), head mass, body mass and musculature vary as a function of species, sub-species, age and biological sex. All of these factors likely affect the initial transfer of energy, as well as the degree of multiplanar motion. Similarly, whereas two straps in conjunction with a bite bar have been used as the traditional restraint device for implementing the HYGE swine injury model^5^ (also see Table 1 from Ref. 18), more recent HYGE studies^8,13^ have migrated to a cable system to better accommodate individual differences in animal morphology. Second, the magnitude of the initial impulsive load and the chosen plane of rotation may also affect results, with lower magnitudes of exposure theorized to result in closer device/head coupling.^18^

The primary aims of the current study were therefore to investigate several factors that may either affect device/head coupling or confound the measurement of head kinematics during large animal acceleration TBI models. Specifically, Experiment 1 determined whether a cable-based restraint device^8,13^ and altered animal body positioning affected device/head coupling relative to the traditional strap system.^5^ Unlike the triaxial angular rate sensor used in our previous study,^18^ Experiment 1 employed a 6 degree of freedom (6DOF) sensor to capture linear acceleration in addition to replicating previous angular velocity measurements. Experiment 2 examined effects associated with a lower targeted angular velocity, with both device and skull data collected on the same acquisition system to more carefully quantify temporal differences in coupling. Finally, we simultaneously compared key head kinematic parameters when the 6DOF sensor was mounted directly to the skull vs. when mounting a triaxial sensor through the scalp in Experiment 3. Experiment 3 therefore ruled out any potential confounds associated with the quantification of head kinematics via a scalp mounted sensor during *in vivo* experiments.

## Materials and Methods

### General Animal Procedures for All Experiments

Animal procedures were approved by our local Institutional Animal Care and Use Committee (IACUC) and the U.S. Army Medical Research & Development Command Office of Research Protections Animal Care and Use Review Office (ACURO). Sexually mature Yucatan or Sinclair swine were fasted but provided *ad libitum* access to water for 6–12 h prior to experimental procedures. Swine were initially sedated with midazolam (0.5 mg/kg IM injection) and pre-medicated with buprenorphine-SR (0.12 mg/kg SC) or buprenorphine (0.02 mg/kg SC or IM). Animals were then intubated and maintained under general anesthesia (isoflurane: 5% induction, 1–4% for maintenance combined with oxygen) with a propofol bolus (0.8–1.5 mg/kg) as needed.

A closed-head TBI was initiated via a pneumatic device (HYGE, Inc., Kittanning, PA, USA) as previously described.^18^ Following an IV bolus of midazolam (0.1–0.5 mg/kg), isoflurane (1–4%) was disconnected approximately 30 s prior to the TBI and immediately re-established post-injury. Device kinematics were quantified via a data acquisition system using an ARS-06 uniplanar angular rate sensor (Applied Technologies Associates, Albuquerque, NM, USA; 25 kHz sampling frequency) that was rigidly mounted to the side arm of the HYGE device. A sensor for measuring head kinematics was positioned on an aluminum mounting plate (details provided in individual experiments). Angular velocity measurements from the device and skull-mounted sensors were smoothed with a 4-pole, Butterworth filter channel frequency class (CFC) 1000 (cut-off frequency = 1650 Hz) based on SAE-J211-1 recommendations.^29^ This was followed by an automated peak identification algorithm to eliminate additional spikes in the data.^18^ A CFC 180 filter (cut-off frequency = 300 Hz), which has been used for previous head impact studies,^4,6,27^ was used to account for increased noise in linear acceleration measurements. All automatically identified peaks were confirmed through visual inspection within each channel, and the peak location manually edited (*N* = 3 across all Experiments) when necessary.

A proxy for trace duration was calculated by measuring full-width at half maximum (FWHM) of the peak value. Both peak angular velocity and FWHM from the resultant velocity trace were used as the primary outcome variables to capture any off-axis rotation (Supplemental Methods). The onset of each kinematic period (device and head) was individually defined based on a sliding window approach. Specifically, the first 1 ms of continuous data points that both exceeded 3 standard deviations from the baseline data collection period and was greater on average than 5 rad/s was selected as the initialization point. The relationship between head and device kinematics was expressed as a ratio (head/device), with 1 equaling a perfect linear coupling and transfer of angular momentum. All animals underwent necropsy to record gross neuropathological findings and maxillofacial fractures (herein defined as nasal, frontal, orbit and mandible bones in the swine), as was done in our previous study.^18^

### Evaluation of Different Restraint Devices (Experiment 1): Detailed Methods

A total of 7 sexually mature Yucatan swine were utilized in Experiment 1. Three Yucatan swine (211.3 ± 6.1 days old; 30.2 ± 1.6 kg; 2 females) were secured to the linkage assembly of the HYGE device with the more traditional restraint device used in most previous HYGE experiments (i.e., two straps connected to a bite bar; Supplemental Fig. 1A). The posterior strap was positioned to be as spatially proximal to the nasium as possible, with the second strap positioned just anterior to first strap. A range of strap sizes were used (1/8 inch length increments) to maximize fit. A modified restraint device (Supplemental Fig. 1B) was used for 4 Yucatan swine (204.8 ± 25.2 days old; 29.5 ± 2.3 kg; 2 female) in which cables were spaced every half inch along the length of the snout.^8,13^ The most posterior cable was positioned as spatially proximal to the nasium as possible. Head kinematic data were lost for one female animal in the cable cohort due to a trigger failure. The data from this animal were therefore excluded from the experiment. Device sensor data from another animal were imputed for descriptive purposes only due to the high reliability (i.e., COV 1–2%) of the HYGE.^18^

Rubber matting (thickness = 6 mm, durometer = 50 A) was placed above the bite bar. Dental epoxy (3M Express^™^ Firm Kit Set) was placed directly under the animal’s upper palette and gauze was packed around the mandible for animals in Experiment 1. These changes in experimental procedures relative to our original study^18^ were implemented in an attempt to both mitigate slippage and provide additional protection against tooth and maxillofacial fractures.^13^ The animal was again positioned such that the center of the cervical spinal column was in a plane slightly above the point of rotation.^18^ The body of the animal was then rotated approximately 70° from ventral recumbency to the right to better align with the final position of the head, theoretically decreasing inertial resistance.

A 6 degree of freedom (6DOF) sensor (Diversified Technical Systems 6DX PRO; 25 kHz sampling rate; 19 × 19 × 14.5 mm, 12 g), which combines a triaxial linear accelerometer and triaxial angular rate sensor, was used in Experiment 1. Our previous study utilized a triaxial angular rate sensor.^18^ The 6DOF sensor was positioned on an aluminum mounting plate (Supplemental Fig. 1C) whose inferior edge was parallel to a plane extending across the most superior aspects of the orbital sockets. Fourteen mm cortical screws mounted the plate to the skull through the scalp. The 6DOF sensor weighed an additional 2 g and was 2.0 mm taller than the triaxial sensor (Diversified Technical Systems ARS3 PRO; 50 kHz sampling rate; 19 × 19 × 12.5 mm, 10 g),^18^ with identical measurements for other dimensions.

### Results: Evaluation of Different Restraint Devices (Experiment 1)

The triaxial and 6DOF sensors were first directly mounted to the restraint device and compared across six phantom shots (i.e., no animals) to determine any differences in sensor recordings. Results (Supplemental Fig. 2; Supplemental Results) demonstrated small but significant differences in FWHM (*p* ≤ 0.001; 6DOF > triaxial), whereas peak angular velocity in the coronal plane was statistically similar (*p* = 0.322).

**Figure 1:**
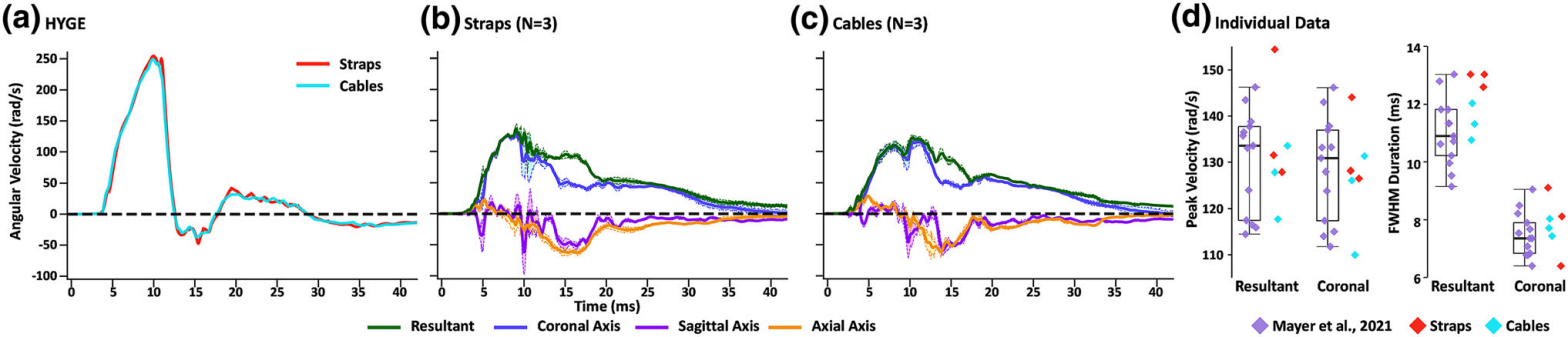
Panel a presents average angular velocity traces (radians per second: rad/s) for HYGE device sensor data from Experiment 1 when animals’ heads were restrained to the bite bar using either straps (red trace) or cables (cyan trace). Panels b and c respectively present average angular velocity for the skull-mounted 6 degree of freedom sensor when straps vs. cables were used as part of the restraint device. Traces for the resultant (green trace) and all three principal axes (coronal = blue trace; sagittal = purple trace; axial = orange trace) are included, with evidence of multiplanar motion in sagittal and axial planes following peak coronal angular velocity. All trace data include estimates of standard error of the mean (lighter colored banding). Panel D displays scatter box plots for key head kinematic parameters including peak angular velocity and full width at half maximum (FWHM) for the resultant and for the coronal plane. Panel D includes previously published^18^ data (*N* = 14; boxplot and lavender diamonds) as a reference point and basis for statistical tests, as well as individual plots for straps (red diamonds; scatter only) and cables (cyan diamonds; scatter only) cohorts.

A primary aim of the current experiment was to determine if differences in restraint devices or animal positioning significantly affected head/device coupling anomalies observed in our previous study.^18^ HYGE sensor data (Fig. 1a; Table 1) was therefore contrasted against combined head kinematic data from the straps (Fig. 1b) and cables (Fig. 1c) cohorts. Results replicated previous findings of significantly decreased peak angular velocity (*t*_4_ = 31.83, *p* < 0.001, *d* = 5.27) and increased duration (FWHM: *t*_4_ = − 14.20, *p* < 0.001, *d* = − 6.98) for the head kinematic relative to device kinematic data. The coupling for peak angular velocity (ratio between the skull-mounted and device sensor data) were qualitatively similar across both restraint devices relative to previous experiments (Mayer *et al.*, 2021^18^ = 0.52; Straps = 0.54; Cables = 0.50). Similarly, trace duration was approximately doubled for the skull-mounted sensor data (Mayer *et al.*, 2021^18^ = 1.89; Straps = 2.25; Cables = 2.01), but was more variable across restraint devices and experimental set-ups compared to peak angular velocity.

**Table 1:** Key parameter results across cohorts from a previous study (Mayer *et al.*, 2021)^18^, Experiment 1 and Experiment 2.

	HYGE (M ± SD)	Head Sensor (M ± SD)			
	Coronal axis	Resultant	Coronal axis	Sagittal axis	Axial axis
Mayer *et al.* (2021) (*N* = 13)					
Peak angular velocity (rad/s)	250.51 ± 3.46	130.22 ± 11.17	128.55 ± 11.36	55.21 ± 14.46	60.04 ± 10.25
FWHM (ms)	5.82 ± 0.13	11.02 ± 1.17	7.50 ± 0.77	–	–
Experiment 1: straps (*N* = 3)					
Peak angular velocity (rad/s)	254.18 ± 3.23	137.92 ± 14.35	132.92 ± 9.69	93.58 ± 40.07	64.19 ± 8.49
FWHM (ms)	5.72 ± 0.11	12.89 ± 0.25	7.88 ± 1.38	–	–
Experiment 1: cables (*N* = 3)					
Peak angular velocity (rad/s)	251.81 ± 2.07^a^	126.37 ± 8.01	122.47 ± 11.14	74.89 ± 21.02	74.82 ± 14.64
FWHM (ms)	5.67 ± 0.06^a^	11.37 ± 0.64	7.73 ± 0.30	–	–
Experiment 2 (*N* = 6)					
Peak angular velocity (rad/s)	168.39 ± 5.19	116.22 ± 7.99	112.54 ± 6.19	85.84 ± 26.52	48.25 ± 6.54
FWHM (ms)	7.95 ± 0.32	13.25 ± 0.38	9.37 ± 2.02	–	–

*M* mean, *SD* standard deviation, *ms* milliseconds, rad/s, radians per second, *FWHM* full width at half maximum

^a^HYGE data for one animal derived using mean imputation

Insufficient power (i.e., more than 20 animals required per cohort) existed to directly compare head kinematics across the two restraint device systems. One-versus-many *t*-tests^17^ were therefore conducted to determine whether each animal in the straps and cables cohorts experienced statistically similar head kinematics as previously published^18^ results (straps as restraint device, no padding or dental epoxy, no rotation of body). Results were not corrected to provide a more liberal threshold for determination of potential differences. Results (Fig. 1d) indicated that the majority of resultant angular velocities (straps *p* range = 0.04–0.38; cables *p* range = 0.08–0.29) experienced by Experiment 1 animals were similar to the previous study despite positional differences and attempts to mitigate slippage. FWHM values (straps *p* range = 0.05–0.06; cables *p* range = 0.10–0.28) also did not meet conventional levels of significance (p < 0.05), but qualitatively appeared to have a different distribution from the initial experiments that varied based on the restraint device.

Results from Experiment 1 also replicated previous findings of complex, multiplanar head motion in the sagittal and axial plane following the peak velocity in the coronal plane (Figs. 1b and 1c; Supplemental Fig. 3; Supplemental Video). Supplemental Fig. 3 provides angular velocity (top row) and linear acceleration (bottom row) data for all animals in the straps (Supplemental Fig. 3A) and cables (Supplemental Fig. 3B) cohort. An average of 21.3 ± 4.2 g of linear acceleration forces was experienced in the sagittal plane at the initiation of the injury using either cables or straps.

Gross necropsy suggested a significant amount of trauma at targeted loads of 250 rad/s. Specifically, results indicated both left (3/3) and right (3/3) maxillofacial fractures that variably involved nasal, orbital and maxillary bones for the strap cohort. Animals with cable restraints exhibited right (2/3) or left (2/3) maxillofacial fractures, as well as right (3/3) mandible fractures. Intracranial hemorrhage was observed in 2/3 strap animals and 1/3 cable animals.

Results from Experiment 1 were suggestive of minimal impact of inertial mass (changes in body position) on peak angular velocity. To further confirm these results, HYGE sensor data were obtained from a convenience sample of 16 Yucatan swine (195.0 ± 35.9 days old; 29.9 ± 3.4 kg; 7 male) and contrasted to matched phantom fires (i.e., without an animal). Animal and phantom data were collected within a maximum of 4 h from each other (mean 123.4 ± 62.4 minutes apart) and used identical pneumatic pressures on the HYGE to maximize comparability. Results from paired *t*-tests (see Supplemental Fig. 4) indicated significantly higher angular velocity between phantom (256.0 ± 6.3 rad/s) and fully loaded (247.6 ± 6.5 rad/s) HYGE exposures (*t*_15_ = 4.84, *p* ≤ 0.001, *d* = 1.33), which lead to the expected significant differences in FWHM (*t*_15_ = − 2.97, *p* = 0.009, *d* = − 0.94; phantom: 5.7 ± 0.2 ms; loaded: 5.9 ± 0.2 ms). Importantly, the ratio of angular velocity between phantom and animal fires was approximately 1.03, suggesting minimal effects of inertial load on the HYGE sensor recordings.

### Coupling at Lower Targeted Angular Peak Velocity (Experiment 2): Detailed Methods

The methodology used in Experiment 2 was nearly identical to Experiment 1. Specifically, a total of 6 sexually mature Yucatan swine (224.3 ± 12.2 days old; 29.4 ± 1.6 kg; 3 females) were used in Experiment 2. All swine were secured to the linkage assembly of the HYGE device using two straps connected to a bite bar. Six mm thick rubber matting (durometer = 50A) was used on the bite bar in conjunction with dental epoxy. Animal positioning was identical to our previous publication,^18^ with the cervical column positioned slightly above the point of rotation and the remainder of the spine in alignment (i.e., no body rotation as was done in Experiment 1). The mounting plate was secured to the skull through the scalp with 14 mm cortical screws, and the 6DOF sensor was again used to measure both angular velocity and linear acceleration. The device sensor was mounted to the side-arm, but the targeted angular velocity was reduced from 250 to 170 rad/s in the coronal plane. Finally, data from both the device and the skull-mounted sensor were now collected on the same data acquisition system to further reduce acquisition confounds. This also permitted the direct comparison of temporal profiles relative to an external trigger.

### Results: Lower Targeted Angular Peak Velocity (Experiment 2)

**Figure 2:**
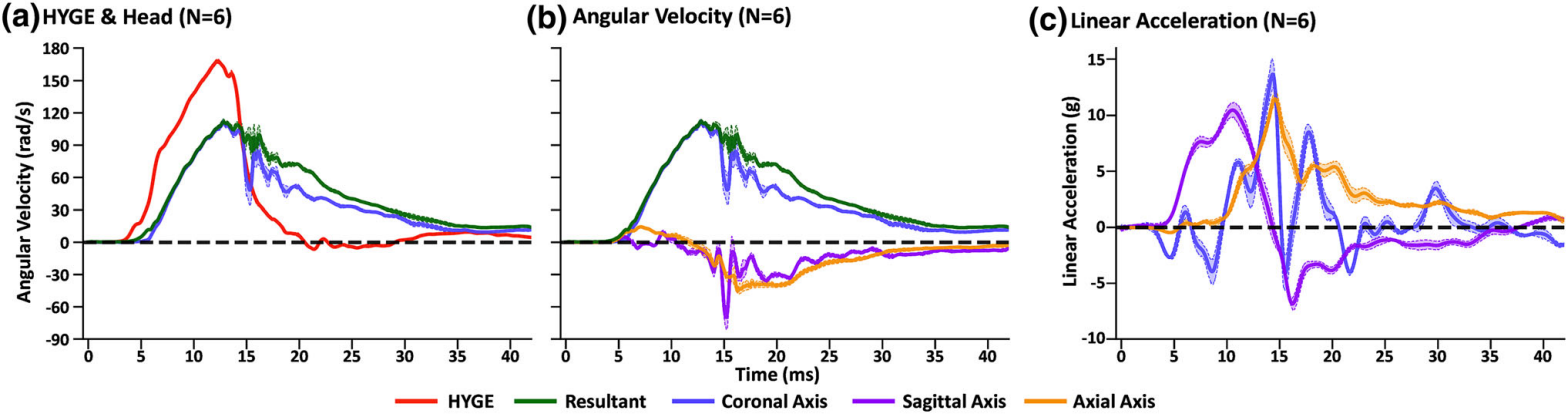
Panel a presents average angular velocity traces (radians per second: rad/s) for HYGE device sensor data (red trace) in addition to resultant (green trace) and coronal (light blue trace) data from the skull-mounted 6 degree of freedom sensor during Experiment 2 (*N* = 6 Yucatan swine). All Experiment 2 data were collected on the same acquisition platform. Panel b includes angular velocity traces for sagittal (purple trace) and axial (orange trace) axes along with the coronal axis and resultant. Evidence of multiplanar motion was again evident for Experiment 2. Panel c displays linear acceleration (g) trace data for the three principal axes. All trace data include estimates of standard error of the mean (lighter colored banding). Angular velocity data were filtered with a 1000 channel frequency class filter, whereas linear acceleration data were filtered with a 180 channel frequency class filter.

Results (Figs. 2a and 2b; Table 1; Supplemental Fig. 5) indicated significantly decreased peak angular velocity (*t*_5_ = − 10.89, *p* < 0.001, *d* = − 7.86) and increased duration (FWHM: *t*_5_ = 25.54, *p* < 0.001, *d* = 15.03) for head relative to device kinematics. However, a closer device-head coupling ratio was evident for Experiment 2 (mean ratio = 0.69) relative to Experiment 1 (mean = 0.53) in terms of peak velocity, as well as duration of kinematics (Experiment 2 = 1.67; Experiment 1 = 2.13). In addition, examination of data based on the initialization time (described in Materials and Methods) indicated a 1.3 ± 0.1 ms temporal delay between the onset of device and head kinematic motion (Fig. 2a).

**Figure 3:**
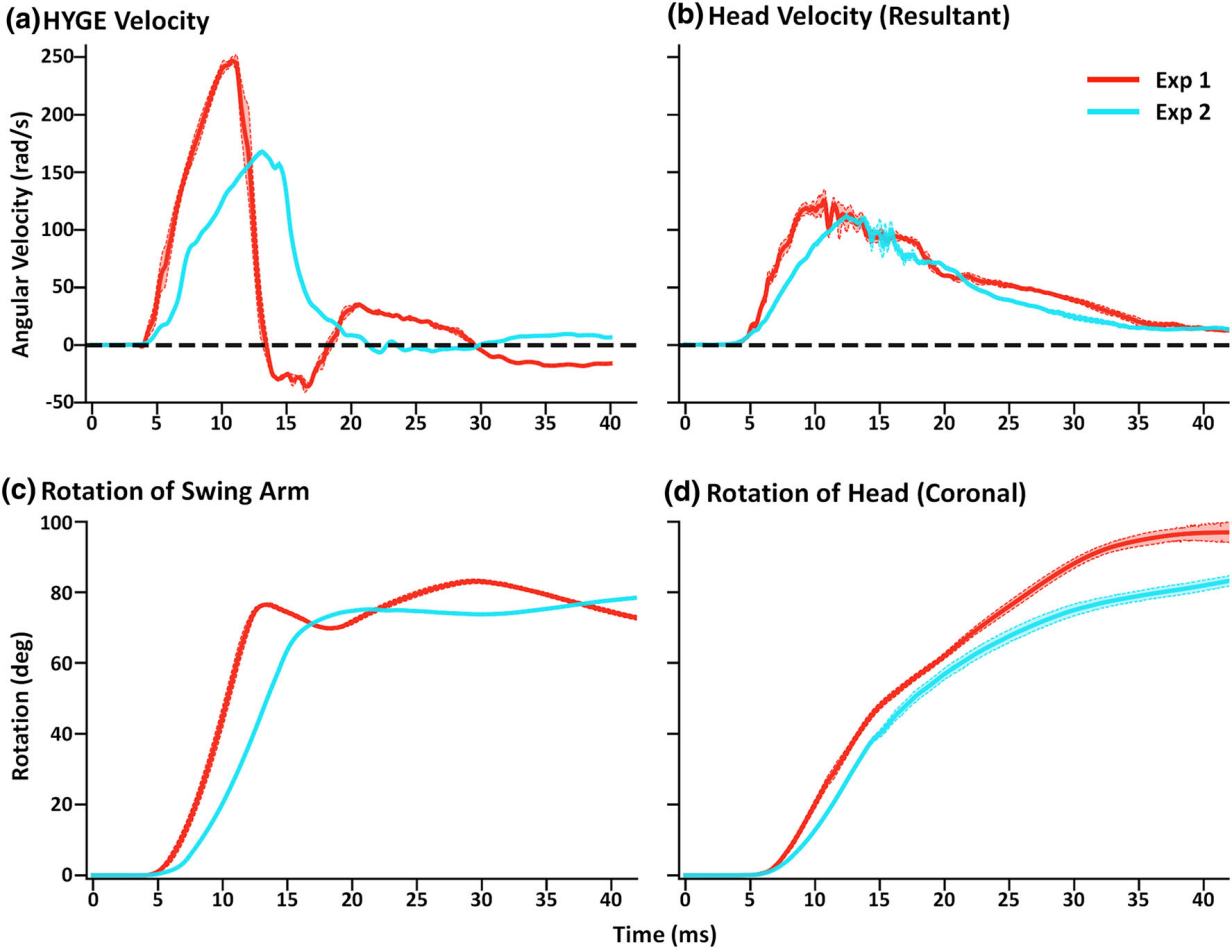
Panel **a** presents average angular velocity (radians/second [rad/s]) trace data from Experiments 1 (Exp 1; red trace with targeted exposure of 250 rad/s) and 2 (Exp 2; cyan trace with targeted exposure of 170 rad/s) for the HYGE sensor. Panel b plots the associated head kinematics from the resultant trace. Data was synchronized between Experiment 1 and 2 for plotting purposes by identifying the start of the first 1 ms window of continuous velocity data that exceeded 3 standard deviations from baseline and had an average greater than 5 rad/s. The bottom row presents average rotational excursion (degrees) over time for the HYGE swing arm (Panel c) and the animal’s head in the coronal plane (Panel d). All trace data include estimates of standard error of the mean (lighter colored banding). These data collectively suggest that while the peak velocity across Experiment 1 and 2 were similar, the rate of change in angular momentum was much higher in Experiment 1 for both device and head kinematics, potentially explaining the more extensive maxillofacial trauma observed in Experiment 1.

Figure 3 respectively plots the device (Panel a) and head (Panel b) kinematic data from Experiments 1 and 2, along with respective angular displacement (Panels c and d). The initialization of each kinematic period was defined using the sliding window approach described in the Materials and Methods section, and temporally locked based on this point with an additional buffering of 4 ms of baseline data. Figure 3 panels A and B demonstrate both the observed differences in peak velocity magnitude and the respective changes in angular acceleration (i.e., slope) in device and head kinematics. Importantly, although peak angular velocity was more similar for head relative to device kinematic data across Experiments 1 and 2, the angular accelerations of both device and head kinematics were much higher during Experiment 1. Collectively, these findings suggest a strong non-linearity between the scaling of device and head kinematics when targeting coronal exposures using the HYGE device.

The next series of analyses compared the head sensor recordings for the resultant motion relative to the plane approximating the coronal axis in Experiment 2. The FWHM of the resultant was longer than the coronal duration (*t*_5_ = 4.95, *p* = 0.004, *d* = 2.29), most likely indicative of multiplanar movement. Head motion in both sagittal (85.8 ± 26.5 rad/s) and axial (48.3 ± 6.5 rad/s) planes again became more evident as following peak angular velocity in the coronal plane. Conversely, there was no statistically significant difference between the resultant and coronal axes for angular velocity (*t*_5_ = 1.49, *p* = 0.20, *d* = 0.50). Supplemental Fig. 5 provides angular velocity (top row) and linear acceleration (bottom row) data for three animals from Experiment 2. Individual and averaged plots (Fig. 1c) indicated approximately half of linear acceleration that was observed in Experiment 1, with 10.8 ± 1.8 g in the sagittal plane at injury initiation.

In contrast to Experiment 1 and previous results, more complex maxillofacial fractures and mandibular fractures were not present in Experiment 2. Specifically, gross necropsy results indicated fractures of only the nasal bones in 5/6 animals (3/6 left, 5/6 right). Intracranial hemorrhage on the dorsal surface of the cerebellum was observed in 2/6 animals.

### Evaluation of Sensor Mounting Techniques (Experiment 3): Detailed Methods

Two fully mature Sinclair (521 and 405 days old; 40.9 and 43.3 kg; both male) swine were used in Experiment 3 to examine potential effects associated with sensor mounting. The Sinclairs had several morphological differences relative to Yucatans including greater mass (head and body), longer and wider snouts, and partially developed canine teeth. The methodology for animal placement (standard sphynx position), restraint device (strap system), a targeted velocity of 250 rad/s in the coronal plane, and sensor placement were identical to our previous study.^18^ The triaxial angular rate sensor was positioned on an aluminum mounting plate with the inferior edge parallel to a plane extending across the most superior aspects of the orbital sockets. Four 14 mm cortical screws were used to secure the mounting plate to the skull through the scalp. In addition, a rectangular area of skin roughly the size and shape of the aluminum mounting plate was excised just posterior to the edge of the first plate. The 6DOF sensor was positioned on a second aluminum plate so that angular velocities could be directly compared across the two mounting procedures. This second plate was secured directly to the skull with four 12 mm cortical screws to account for differences in the depth of the scalp. The posterior edge of the first plate and anterior edge of the second plate were aligned in parallel as closely as possible (distance between bottom edges of plates = 4.3 or 3.5 mm, top edges = 8.4 or 6.2 mm across the two animals).

### Results: Sensor Mount Technique (Experiment 3)

As expected (Supplemental Fig. 6A and 6B), there were reductions in angular velocity and increased FWHM between the HYGE and the skull-mounted sensor in the Sinclair swine. The magnitude of these reductions were larger in Experiment 3 than previous studies (see Discussion section), resulting in a lower head/device coupling for both angular velocity (Scalp=0.26; Direct=0.26) and FWHM (Scalp=3.30; Direct=3.34). Critically, Supplemental Fig. 6B demonstrates that there were no differences between the resultant angular velocity from the triaxial and 6DOF sensors when respectively mounted through the scalp or directly into the skull.

## Discussion

Rapid acceleration/deceleration of the head represents a common factor in human TBI, and is best studied in large animal models with gyrencephalic brains.^10,28,33^ Identifying potential differences between head and device kinematics due to coupling discrepancies is critical for establishing injury thresholds, especially when scaling these thresholds across multiple species.^2,9,10^ From a biomechanical perspective, the HYGE has traditionally been considered to be a closed system that transfers angular momentum between the restraint device and the swine head in a linear fashion. However, our previous study demonstrated an unexpected ~ 50% reduction in angular velocity for head vs. device kinematics in conjunction with an approximate doubling of the duration during a targeted 250 rad/s coronal rotation, as well as evidence of multiplanar motion.^18^ These differences in head/device coupling were replicated in the current study across two different restraint devices with additional alterations in animal positioning. New results further suggest minimal amounts of linear acceleration with HYGE exposures (~ 10 to 20 g across Experiments 1 and 2), commensurate with the peak head kinematics experienced during the purposeful heading of a soccer ball.^21,32^

Results from Experiment 1 suggest that the coupling between device and head kinematics was not significantly affected relative to previous results^18^ by either altering animal body positioning to potentially reduce inertial resistance, or through the use of additional materials in the restraint device (rubber matting and dental epoxy) to mitigate slippage. Separate analyses with a convenience sample of Yucatan swine (*N* = 16) indicated small but statistically significant reductions (approximately 3%) in device angular velocity when the impulsive load was delivered with or without an animal in the restraint device. These results further substantiate that the inertial mass associated with the swine head/body has little impact on the *device* kinematics at high angular velocities (e.g., 250 rad/s).

Experiment 1 also compared the traditional strap set-up for the restraint device^5^ to the recently developed cable system.^8,13^ Similar to our previous study,^18^ both restraint devices resulted in similar ~ 50% reductions in peak angular velocity and a doubling of the duration for head relative to device kinematics. It is notable that a multitude of restraint device variations, animal positioning, and other mitigation strategies can theoretically be attempted to increase head/device coupling and reduce slippage. Current results should therefore not be considered definitive. However, this testing approach may not be financially or ethically feasible in large animal models of TBI,^33^ as power analyses indicated that much larger samples (*N* ≥ 20 animals per cohort) would be necessary to establish potential statistical difference in key kinematic parameters between the two tested restraint devices. Nonetheless, findings suggest that the *tested* mitigations for slippage and animal positioning were of insufficient magnitude to result in statistically significant differences in head/device coupling relative to our previous study^18^ at current sample sizes. Additional results from Experiment 1 also suggested that inertial mass was not a large contributor to angular speeds observed on the HYGE device for coronal exposures at 250 rad/s.

Experiment 1 findings of a reduction in peak angular velocity in conjunction with increased temporal duration could be construed as a conservation of energy according to basic Newtonian laws. However, this view may be overly simplistic in the case of the HYGE. In Experiment 2, a lower targeted exposure (170 rad/s) in the coronal plane resulted in a closer coupling between device/head kinematics, as well as a delay in head kinematic onset relative to device. Specifically, a reduction of approximately 80 rad/s (32%) in device kinematics from Experiment 1 to Experiment 2 resulted in a reduction of only 19 rad/s (14%) in terms of measured head kinematics (Table 1) and therefore a closer coupling between head/device kinematics (69%) relative to the 250 rad/s exposures. Importantly, even though the magnitude of the angular velocity was only reduced by 19 rad/s, a comparison of the trace suggested a greater and more rapid change in angular velocity for Experiment 1. These findings highlight the importance of directly quantifying both device and head kinematics, and empirically establishing how they couple at different loading factors. A lack of accuracy in head kinematic characterization may more adversely affect the accuracy of injury threshold criteria and finite element modeling algorithms^2,9^ relative to studies characterizing injury pathology.

**Figure 4:**
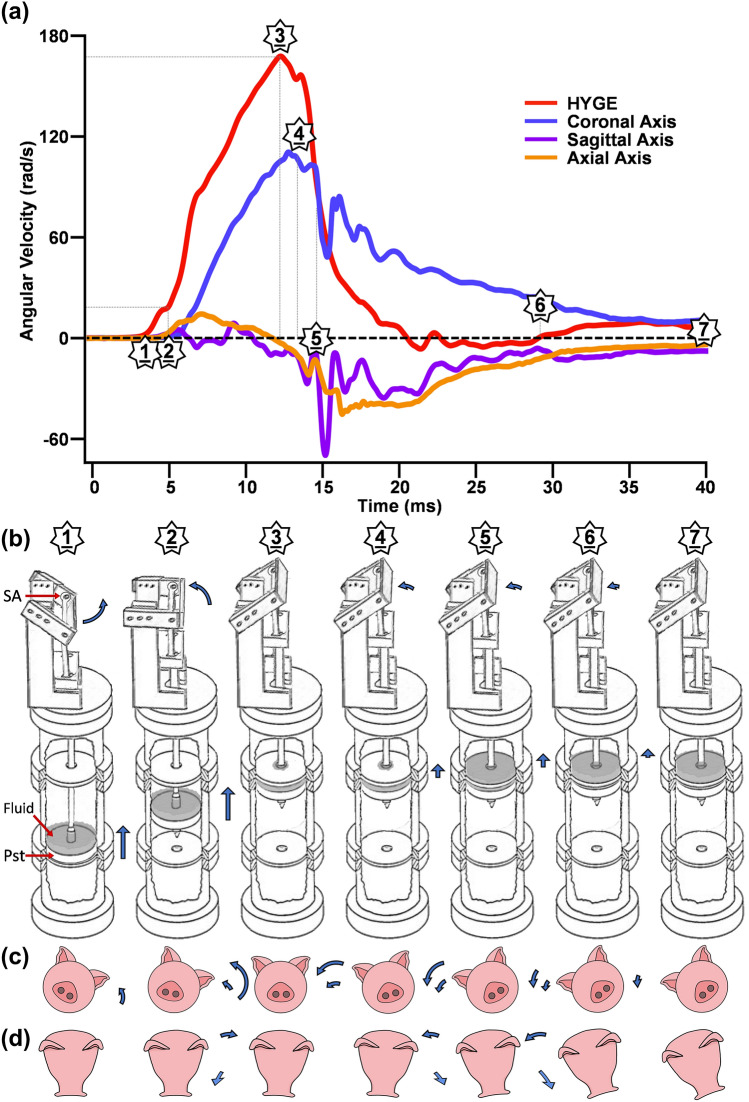
Figure 4 presents a theoretical depiction of the events that subsequently produce device and head kinematics for HYGE experiments. Panel a presents Experiment 2 average angular velocity traces (radians per second: rad/s) for both the HYGE device (sensor mounted to swing arm [SA]; red trace) and 6 degree of freedom sensor when mounted to the skull (coronal = blue trace; sagittal = purple trace; axial = orange trace). Panel b presents the primary drivers of linear and angular velocity in the HYGE pneumatic device, including a fluid barrier (Fluid) that sits upon the piston (Pst). Panels c (coronal movement) and d (out-of-plane movement) present angular rotation of the swine head during a coronal exposure as a function of time. The change in position between phases for the device components (Panel b) and the swine skull (Panels c and d) is indicated with blue arrows, with changes in arrow size depicting theoretical changes in linear or angular velocity magnitude. For Panel c, the outer ring arrows depicts theoretical rotation of the skull while the inner ring arrow shows rotation of the snout. A full description of each theoretical phase is presented in the Discussion section.

As previously described,^5^ the HYGE converts linear momentum (piston) to angular momentum through the device swing arm and rigidly connected restraint device (Fig. 4). However, the maxillary structures (teeth, gums and palate) of the swine are likely not to be perfectly aligned with the restraint device, even with the use of materials that are individually molded for each animal to better secure the snout to the restraint device fit.^8,13,18^ There is subsequently a collision between the restraint device (principally the bite bar) and the swine’s maxillofacial structures, in which the restraint device must first overcome the inertial mass of the swine head (principle) and body (secondary) prior to the transfer of angular momentum to the swine head. This initial transfer of angular momentum may have resulted in the slight inflection point observed in the HYGE angular velocity trace at approximately 4–5 ms in Fig. 4, which coincides with the start of the multiplanar swine head motion.

In the current series of studies (coronal exposure with a rightwards rotation), the initial mechanical coupling initially occurs between the left maxillofacial bones and the opposing right mandible. The differential pattern of injury observed between Experiments (Experiment 1 > 2) indicates that maxillofacial structures absorb the energy from the restraint device inducing deformation. This could lead to fractures at higher levels of angular velocity in association with a decreased rate of transferred angular momentum, similar to crumple zones in motor vehicle collisions. Regardless of the nature of the fracture, the tissue deformation during the initial transfer of momentum fundamentally delays the onset of the head kinematics (Fig. 4b, Phase 1). After a delay, the angular velocity of the restraint device and the swine head begin to physically couple in a more dependent fashion (Phase 2), as demonstrated by the increasing angular velocity observed for the skull sensor data in the coronal plane.

The angular velocity of the restraint device peaks (Phase 3 = apex of trace) and then experiences a rapid deceleration as the metering pin of the piston enters the deceleration orifice and the piston encounters the physical barrier of hydraulic fluid. The swine head maintains a similar angular velocity even as the device rapidly decelerates (Phase 4), which may be attributable to the large differences in initial angular velocity and angular momentum of the swine head. The angular velocity of the swine head then decreases in the coronal plane as it becomes fully loaded against the straps/cables of the decelerating restraint device (Phase 5). The kinetic energy of the swine head now becomes quasi-independent of the restraint device (see brief increase in coronal velocity) and is further dissipated through complex multiplanar motion in both the axial and sagittal planes. Meanwhile, the piston slowly compresses the remainder of the hydraulic fluid through the deceleration orifice (Phase 6), such that the restraint device now regains momentum in the same positive arc but at a very low velocity before coming to a complete stop (Phase 7). This transition is also associated with very minimal, low energy movement of the head.

Thus, the observed differences in head vs. device kinematics in the current and previous study^18^ most likely resulted from a delay in the initial coupling between the restraint device and maxillary structures. This is further compounded by tissue deformation in maxillofacial structures, and by the extremely brief nature of the linear trajectory of the HYGE piston before it is terminated by the hydraulic fluid barrier. A previous study^20^ may therefore have observed a closer coupling between the HYGE device and a swine skull due to the material properties of specimen (i.e., swine skull mounted inside of a rigid metallic can). The restraint device and the swine head therefore start off with different velocities and angular momentums, which may be a critical consideration given that injury threshold may primarily be defined by changes in velocity rather than acceleration based on the brief duration of the HYGE event.^10,14,22^ Although preliminary, current findings suggest that the initial coupling between the device/head may principally drive injury rather than the decelerative phase based on targeted peak angular velocities during coronal exposures.

Multiple methodologies have been used in previous cadaver studies for mounting sensors to the skull (see Supplemental Table 1), including direct sensor mounting to the skull vs. mounting to the skull through the scalp.^36,37^ Experiment 3 suggests nearly equivalent head kinematics for these two mounting procedures in a small sample of animals (*N* = 2). This mitigates the likelihood of complex interactions between the sensor, mounting plate, scalp and skull as confounds for non-linear coupling between the device and head.^18^ Therefore, the biopsy punch and scalp mounting may be the preferred method for future large animal studies that seek to directly measure head kinematics given the less invasive nature of the procedure relative to excising a large portion of the scalp, particularly for survival studies.

Experiment 3 used two fully mature, Sinclair male swine. Experiments 1 and 2, as well as our previous study,^18^ all used 5–7 month old Yucatan swine, corresponding to initial sexual maturity. Results indicated that the head kinematics experienced by the Sinclairs was approximately 50–70 rad/s, much lower than the average angular velocity observed with Yucatan swine under identical loading conditions (i.e., targeted rotation of ~ 250 rad/s in the coronal plane). The fully matured Sinclair swine exhibited several morphological differences relative to Yucatan swine including larger heads, larger body mass, more muscular necks, longer and wider snouts, and more developed canine teeth (including bony protuberances in the upper palate). The longer snout causes the center of the head (and sensor placement) to be positioned further from the restraint device, and differences in the morphology of maxillary structures could critically impact on the delay in energy transfer from the bite bar to the head. The sample size employed in Experiment 3 was too small to disambiguate the multitude of factors that may influence head/device coupling across swine subspecies, as the primary rationale for the study was to compare sensor mounting techniques. However, these results further reinforce the importance of direct measurement of head kinematics in acceleration injury models, and the challenges associated with extrapolating results from one species or even subspecies to the next, as well as across different developmental stages and biological sex.

The primary limitation of the current series of experiments was the relatively small sample sizes. This prohibited the examination of such factors as the role of biological sex in head/device coupling, how biological sex may affect maxillofacial fractures, and potential differences between restraint systems. Importantly, sample sizes were sufficient to reproduce the non-linear nature of head/device coupling,^18^ a necessary first step for inter-laboratory studies to determine the precision of testing methods.^30^ Large animal studies must also more carefully factor the ethical and financial considerations associated with higher-order species against the amount of knowledge gained. A second limitation was that the current sensor placement measured the kinematics of the skull rather than the brain itself, with known differences in parenchymal dynamics and deformations occurring during injury.^2,9,10,31^ Third, although the triaxial and 6DOF sensors were calibrated for the main variable of interest (angular velocity), small differences existed for other kinematic parameters (duration), likely as a result of different physical dimensions of the sensors. Although small, these differences may have impacted on the comparison of data from the current and previous studies.^18^

A final limitation was that the current series of experiments focused on angular velocity rather than angular acceleration as the main outcome measure similar to previous studies.^5^ Due to the errors associated with numerical differentiation of angular velocity to calculate angular acceleration,^3^ angular acceleration was not assessed in the current study. Future studies could consider using arrays of nine accelerometers or six accelerometers and three angular rate sensors to algebraically solve for angular acceleration.^12^

In summary, current results suggest that the coupling between device and head kinematics in the coronal plane vary based on the initial impulsive load (lower angular velocities over longer durations may increase coupling), with the initial device load having a greater effect on angular momentum rather than the peak angular velocity of the head. This relationship is partially dependent on mechanical properties of the HYGE (travel distance of the piston, amount of fluid, *etc*.), and likely varies as a function of both species-related and individual differences in snout and head morphology. Preliminary results also suggest that increased angular velocity of the device appears to be correlated with the amount of observed maxillofacial trauma. Future studies are required to determine the relationship between head and device kinematics in other planes of rotation (axial and sagittal) and at other impulsive loads, as well as to determine how pathology and biomarker expression varies as a function of head kinematics for milder injuries. Current findings suggest that direct quantification of head kinematics is warranted to empirically establish actual injury loads experienced in most experimental models.

## Supplementary Information

Below is the link to the electronic supplementary material.

Supplementary file1 (PDF 1301 kb)

Supplementary file2 (MP4 49337 kb)
