# Supplementary material for: Non-Linear Device Head Coupling and Temporal Delays in Large Animal Acceleration Models of Traumatic Brain Injury
Source: Ann Biomed Eng. 2022 Apr 2;50(6):728–39. doi: 10.1007/s10439-022-02953-w (PMC9079018; doi:10.1007/s10439-022-02953-w)
Supplement: Supplementary file 1 — Supplementary file1 (PDF 1301 kb) [file 10439_2022_2953_MOESM1_ESM.pdf]

Supplemental Methods

Resultant angular velocity of the head was calculated by squaring and summing angular velocity components from the three principal axes of rotation. Peak resultant angular velocity and full-width at half maximum (FWHM) for the resultant were used as the primary outcome variables. FWHM was a proxy measure for duration, and was calculated by estimating the width (unit = milliseconds) of the impulse at the peak duration. FWHM was chosen given the more complex and longer kinematics experienced by animals relative to the device sensor (see Figure 1A relative to 1B and 1C). The mounted sensor experienced off-axis rotation (i.e., plane of skull near orbital sockets is sloped downward), which varied owing to differences in individual animal morphometry. The resultant therefore represents a surrogate of both uniplanar and multiplanar head motion, as well as addressing individual differences in mounting procedures. All effects sizes were calculated using the method recommended by Morris and Deshon[1] to account for shared variance.

Supplemental Results for Experiment 1

Paired sample t-tests were implemented to investigate potential differences among key kinematic parameters between the 6 degree of freedom (6DOF) and triaxial skull sensors when simultaneously mounted onto the bite bar of the HYGE device over six phantom shots (no swine). Although qualitatively similar (Supplemental Figure 2), statistical results indicated significantly reduced FWHM (t_5=16.57, p≤0.001, d=10.22) and time to peak (t_5= 25.33, p≤0.001, d=16.23) for the triaxial sensor relative to the 6DOF sensor. Conversely, no significant pairwise difference was observed for peak velocity

(p=0.322; d=-0.74). These findings suggests small differences in recorded kinematics of the sensor alone, likely a result of the different sensor weights.

References

1. Morris, S. B., R. P. DeShon. Combining effect size estimates in meta-analysis with repeated measures and independent-groups designs. Psychol Methods 7:105-125, 2002.
2. McIntosh A. S., D. Kallieris, R. Mattern, and E. Miltner. Head and neck injury resulting from low velocity direct impact. SAE Technical Paper; 1993.
3. Pintar, F. A., N. Yoganandan, A. Sances, and J. F. Cusick. "Experimental Production of Head-Neck Injuries Under Dynamic Forces." In: Head and Neck Injuries in Sports, ASTM STP 1229, edited by Head and Neck Injuries in Sports, ASTM STP 1229. Philadelphia, PA: ASTM International, 1994, pp.
4. Hardy, W. N., C. D. Foster, M. J. Mason, K. H. Yang, A. I. King, and S. Tashman. Investigation of head injury mechanisms using neutral density technology and high-speed biplanar X-ray. Stapp Car Crash J 45:337-368, 2001.
5. Yoganandan, N., J. Zhang, and F. Pintar. Force and acceleration corridors from lateral head impact. Traffic Inj Prev 5:368-373, 2004.
6. Depreitere, B., L. C. Van, J. V. Sloten, A. R. Van, G. Van der Perre, C. Plets, and J. Goffin. Mechanics of acute subdural hematomas resulting from bridging vein rupture. J Neurosurg 104:950-956, 2006.
7. Yoganandan, N., J. Zhang, F. A. Pintar, and L. Y. King. Lightweight low-profile nine-accelerometer package to obtain head angular accelerations in short-duration impacts. J Biomech 39:1347-1354, 2006.

8. Hardy, W. N., M. J. Mason, C. D. Foster, C. S. Shah, J. M. Kopacz, K. H. Yang, A. I. King, J. Bishop, M. Bey, W. Anderst, and S. Tashman. A study of the response of the human cadaver head to impact. Stapp Car Crash J 51:17-80, 2007.
9. Wu, L. C., K. Laksari, C. Kuo, J. F. Luck, S. Kleiven, C. R. 'Dale' Bass, and D. B. Camarillo. Bandwidth and sample rate requirements for wearable head impact sensors. J Biomech 49:2918-2924, 2016.

Supplemental Table 1. Sensor mounting in cadaver studies.

Study	Instrumentation	Mount	Anchors	Instrument Location	Figures/ Schemata
McIntosh et al. (1993)[2]	Primary: Cubic 3-2-2-2 accelerometer array; Secondary: Triaxial accelerometer	Mounting plate	Not reported	Primary: Mid-sagittal plane, top of head; Secondary: Clivus during occipital impacts, Posterior aspect of T1 during lateral impacts	Yes
Pintar et al. (1994)[3]	Primary: Triaxial accelerometer; Secondary: 2 uniaxial accelerometers	Not reported	PMMA	Primary: Temporo-parietal bone of head; Secondary: Mastoid process and antero-lateral aspect of C4	No
Hardy et al. (2001)[4]	Tetrahedral 3-2-2-2 accelerometer array	Metal interface	3 screws and PMMA	Mid-sagittal plane, top of head	Yes
Yoganandan et al. (2004)[5]	Three triaxial accelerometers (three uni-axial accelerometers affixed to metal cube)	Contoured plates	Screws at each corner	At the contralateral temporo-parietal impact site and at anterior and posterior regions of the cranium	Yes
Depreitere et al. (2006)[6]	Three uniaxial accelerometers	16 cm curved aluminum profile bar	Screws for profile to bone, wax for sensor to profile	Superior-to-inferior line along left side of head behind ear	Yes
Yoganandan et al. (2006)[7]	Primary: Tetrahedral aluminum 3-2-2-2 accelerometer array; Secondary: Three triaxial accelerometers	None, direct to bone	6 orthopaedic screws	Primary: Right temporo-parietal surface of skull; Secondary: Vertex, frontal, and occipital regions along midline	Yes
Hardy et al. (2007)[8]	Tetrahedral 3-2-2-2 accelerometer array	Nylon pedestal	Polyester resin	Facial cavity	Yes
Wu et al. (2016)[9]	Triaxial accelerometer and triaxial gyroscope block	Not reported	Not reported	Occipital bone	Yes

T1 = first thoracic vertebra; C4 = fourth cervical vertebra; PMMA = polymethyl-methacrylate, an acrylic glass

Supplemental Video: This video depicts the average of the angular rotation (degrees [deg]) over time experienced by animals in Experiment 1 for the cohort restrained to the bite bar with straps (left side) and the cohort restrained to the bite bar with cables (right side). Data from the three principal axes (coronal: blue, axial: purple, sagittal: orange) are displayed. The swine head used in the panels is derived from an MRI collected in a previous study, but is representative of the species and strain (Yucatan) used in Experiments 1 and 2. Playback is at 1/200 of normal speed.

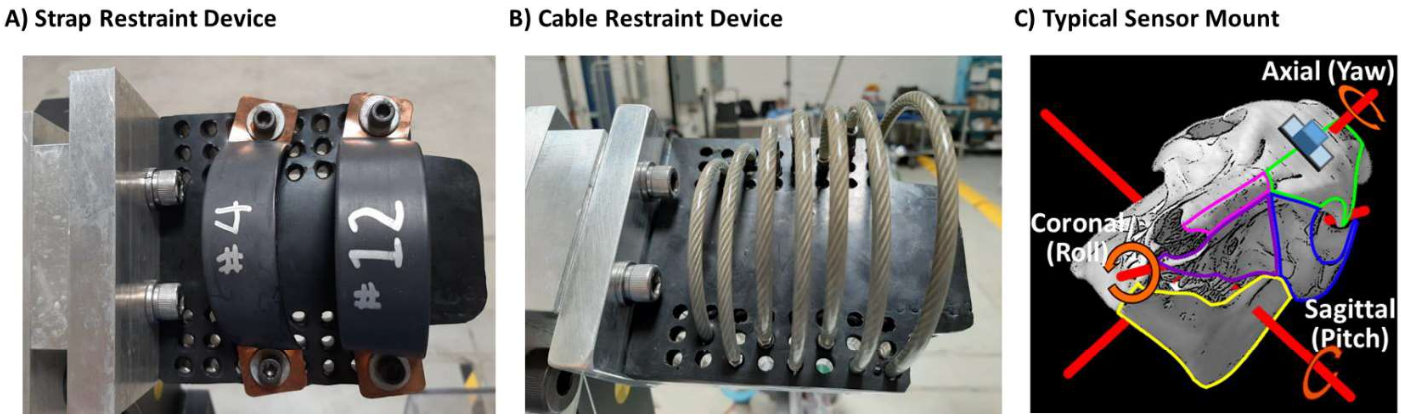

Supplemental Figure 1: This figure depicts the restraint devices used in Experiment 1, including the bite bar with either straps (Panel A) or cables (Panel B) along with rubber matting. Panel C shows a representative pig skull, and demonstrates the three principal rotational axes (red rods). The skull is rotated 44° along the coronal axis to match the initial starting point of the skull in all three experiments. In addition, the panel shows placement of the skull sensor (blue cube) and plate (grey rectangle), as well as typical areas for fractures (green outline: frontal bone; blue: orbital bone; pink: nasal bone; purple: maxilla; yellow: mandible). Panel C picture adapted from skull scanned by the University of Texas High-Resolution X-ray CT Facility (NSF IIS-0208675) located at http://digimorph.org/specimens/Sus_scrofa/skull/.

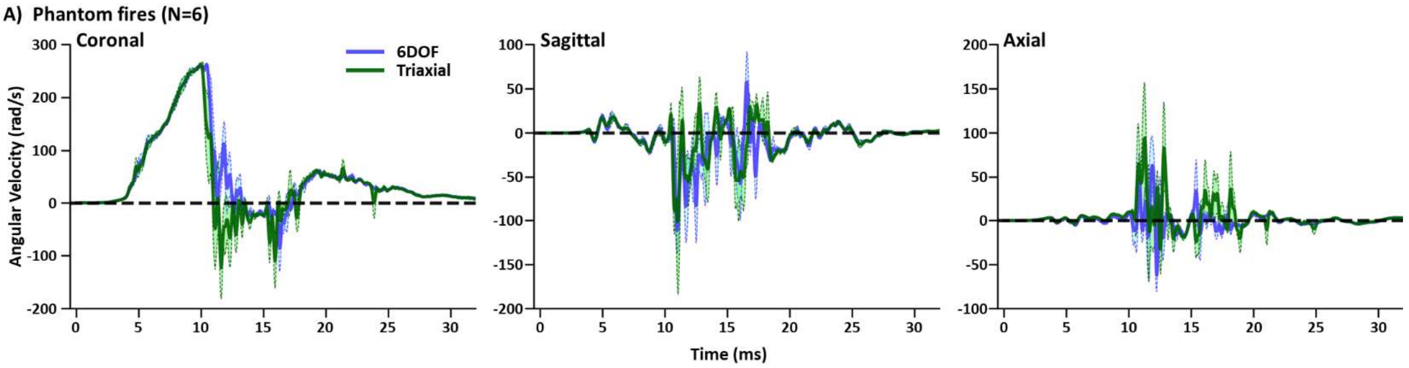

Supplemental Figure 2: This figure presents average angular velocity traces (radians per second: rad/s) for the 6 degree of freedom (6DOF; blue trace) and triaxial (green trace) sensors when mounted directly to the bite bar during 6 phantom fires (no animal present). Data are presented separately for all three principal axes with scale adjusted to maximize comparisons within each axis. Results indicate a longer duration with the 6DOF sensor, most likely a result of the increased weight.

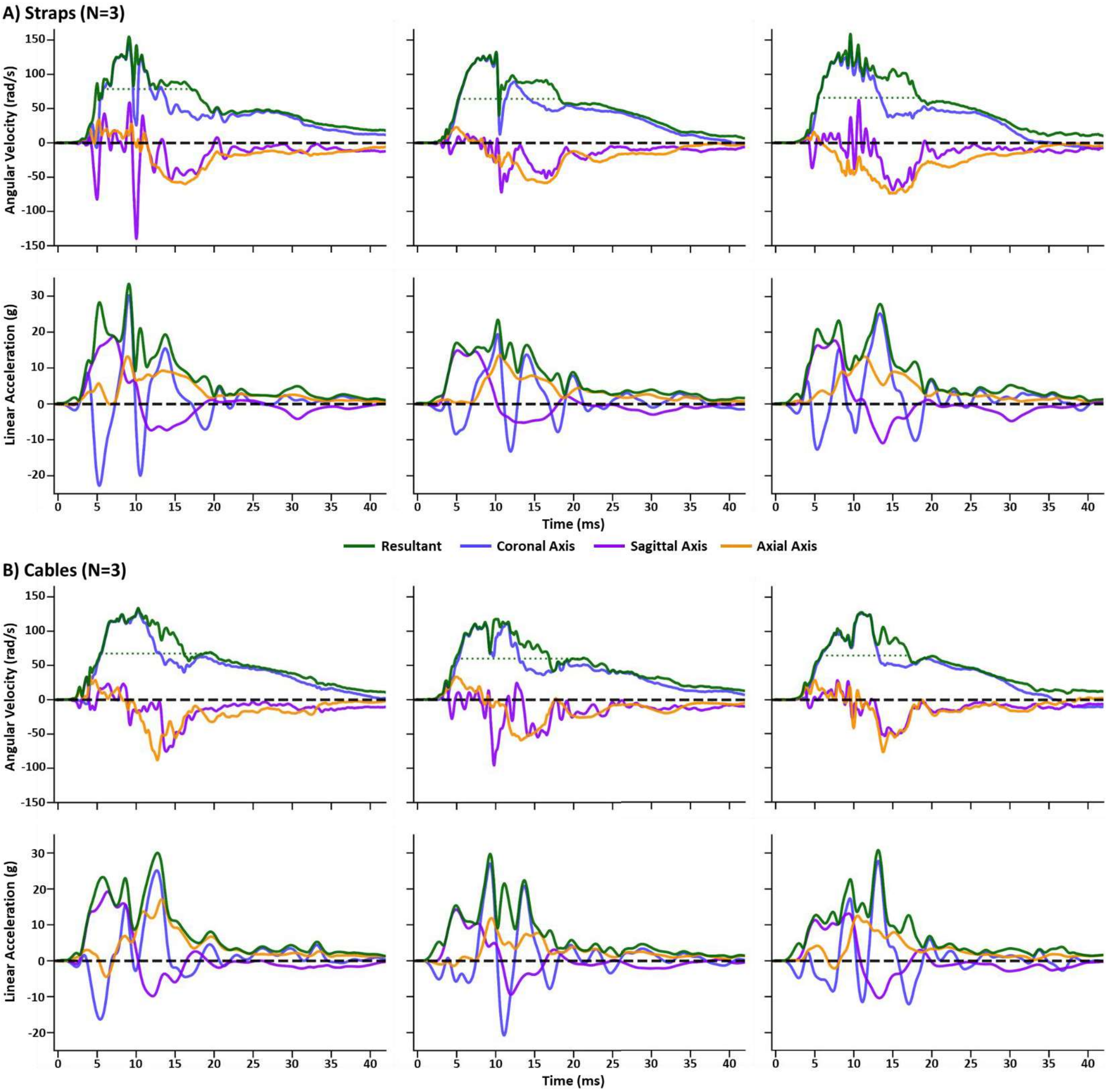

Supplemental Figure 3: Panel A presents data collected with the six degree of freedom sensor during Experiment 1 for all three animals from the straps (Panel A) and cables (Panel B) cohort. The top row of each panel depicts angular velocity traces (radians per second [rad/s]), while the bottom row depicts linear acceleration (g) trace data. Resultant

data (green trace) as well as data for all three principal axes (coronal = blue trace; sagittal = purple trace; axial = orange trace) are presented. In angular acceleration data, the full-width half-maximum of the resultant is depicted with a dotted green line, spanning the first and last point at which the trace is above said height. Although slight differences are observed across the individual animals, the pattern of recorded head kinematics are consistent for coronal, sagittal and axial planes. Angular velocity data were filtered smoothed with 1000 channel frequency class filter, whereas linear acceleration data were filtered with a 180 filter.

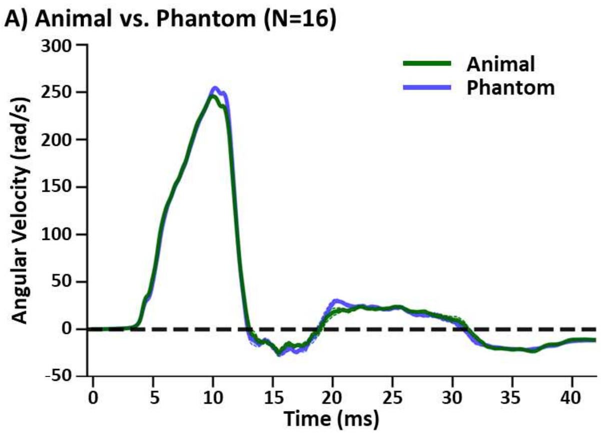

Supplemental Figure 4: This figure presents average angular velocity traces (radians per second: rad/s) of temporally proximal (collected within 4 hours of each other) pairs of data for the HYGE swing arm sensor when fired either with an animal (Yucatan swine; N=16) mounted to the restraint device (green trace) or during a phantom shot (i.e., no animal present; blue trace) under identical initial loading conditions. These results demonstrate a small but significant difference for peak angular velocity on the HYGE swing arm due to inertial resistance from swine mass.

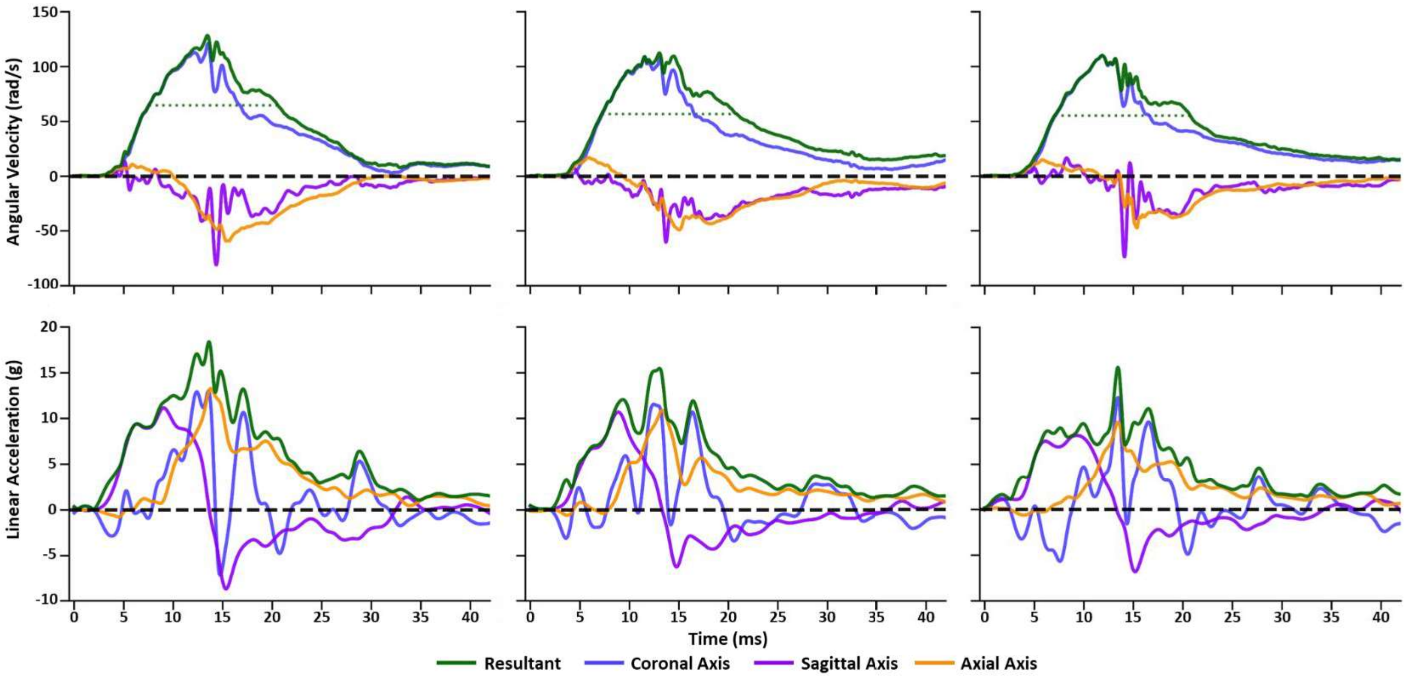

<u>Supplemental Figure 5:</u> Panel A presents data collected with the six degree of freedom sensor during Experiment 2 for three selected animals (N=6). The top row depicts angular velocity traces (radians per second [rad/s]), while the bottom row depicts linear acceleration (g) trace data. Resultant data (green trace) as well as data for all three principal axes (coronal = blue trace; sagittal = purple trace; axial = orange trace) are presented. In angular acceleration data, the full-width half-maximum of the resultant is depicted with a dotted green line, spanning the first and last point at which the trace is above said height. Angular velocity data were filtered smoothed with 1000 channel frequency class filter, whereas linear acceleration data were filtered with a 180 filter.

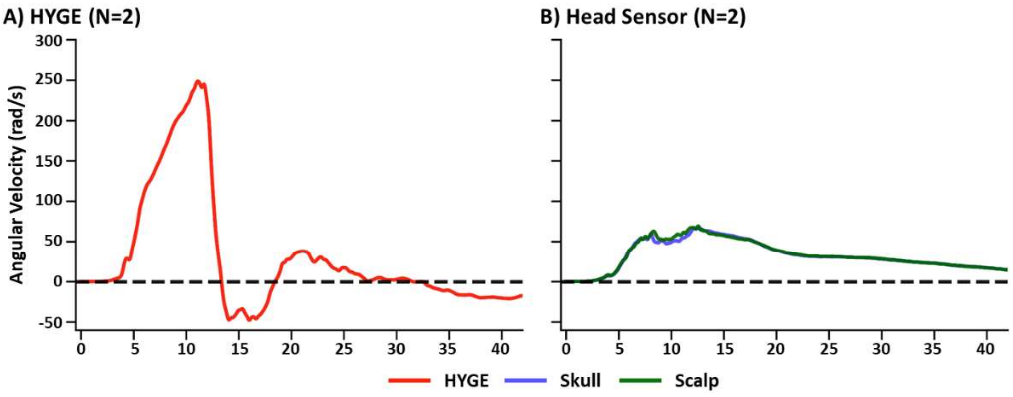

Supplemental Figure 6: This figure presents average angular velocity traces (radians per second: rad/s) for sensor data when mounted to either the HYGE (Panel A; red trace), directly to the skull (Panel B; blue trace; 6 degree of freedom sensor) or to the skull through the scalp (Panel B; green trace; triaxial sensor) for 2 Sinclair swine. Panel B includes data for the resultant only, and demonstrates nearly identical profiles regardless of skull mounting methodology.
